# Validity and test-retest reliability of manual goniometers for measuring passive hip range of motion in femoroacetabular impingement patients.

**DOI:** 10.1186/1471-2474-11-194

**Published:** 2010-08-31

**Authors:** Silvio Nussbaumer, Michael Leunig, Julia F Glatthorn, Simone Stauffacher, Hans Gerber, Nicola A Maffiuletti

**Affiliations:** 1 Neuromuscular Research Laboratory, Schulthess Clinic, Zurich, Switzerland; 2 Institute for Biomechanics, ETH Zurich, Switzerland; 3 Hip Service, Schulthess Clinic, Zurich, Switzerland

## Abstract

**Background:**

The aims of this study were to evaluate the construct validity (known group), concurrent validity (criterion based) and test-retest (intra-rater) reliability of manual goniometers to measure passive hip range of motion (ROM) in femoroacetabular impingement patients and healthy controls.

**Methods:**

Passive hip flexion, abduction, adduction, internal and external rotation ROMs were simultaneously measured with a conventional goniometer and an electromagnetic tracking system (ETS) on two different testing sessions. A total of 15 patients and 15 sex- and age-matched healthy controls participated in the study.

**Results:**

The goniometer provided greater hip ROM values compared to the ETS (range 2.0-18.9 degrees; *P *< 0.001); good concurrent validity was only achieved for hip abduction and internal rotation, with intraclass correlation coefficients (ICC) of 0.94 and 0.88, respectively. Both devices detected lower hip abduction ROM in patients compared to controls (*P *< 0.01). Test-retest reliability was good with ICCs higher 0.90, except for hip adduction (0.82-0.84). Reliability estimates did not differ between the goniometer and the ETS.

**Conclusions:**

The present study suggests that goniometer-based assessments considerably overestimate hip joint ROM by measuring intersegmental angles (e.g., thigh flexion on trunk for hip flexion) rather than true hip ROM. It is likely that uncontrolled pelvic rotation and tilt due to difficulties in placing the goniometer properly and in performing the anatomically correct ROM contribute to the overrating of the arc of these motions. Nevertheless, conventional manual goniometers can be used with confidence for longitudinal assessments in the clinic.

## Background

Hip joint range of motion (ROM) is a basic clinical parameter for diagnosing hip diseases, such as osteoarthritis [1,2] or femoroacetabular impingement (FAI) [3,4], and for monitoring the efficacy of a treatment [5]. Hip joint ROM is widely assessed using low-technology tools such as manual goniometers or inclinometers. The advantages of goniometry are the simplicity in assessing ROM, the direct measurement of joint angles without any data reduction process and the low cost of the instrument. The two-arm goniometer is still the most commonly used, economical and portable device for the evaluation of ROM [6], despite acknowledged limitations. Major drawbacks of goniometry are that the starting position, the center of rotation, the long axis of the limb and the true vertical and horizontal positions can only be visually estimated; moreover, conventional goniometers must be held with two hands, leaving neither hand free for stabilization of the body or the proximal part of the joint [6]. There are also difficulties in monitoring joints that are surrounded by large amounts of soft tissue, such as the hip [7]. In addition, manual goniometers assess joint flexibility only in two dimensions; however, as most of the hip ROM measures in clinical practice are practically in-plane movements, this limitation is minor. The validity (i.e., the degree to which a measurement actually measures what it claims to measure) and reliability (i.e., the degree to which a measurement is consistent and stable) of manual goniometers have therefore been questioned, especially for measuring hip flexion. Bohannon et al. [8] showed that in the hip flexion movement, as measured in a clinical setting, more than a quarter of the ROM can be attributed to pelvic tilt, leading to an immense misinterpretation of this movement due to the insensitivity of manual goniometers for secondary pelvic movement. Elson and Aspinall [9] proposed a new method for measuring range of hip flexion by palpating the lumbosacral junction to allow early identification of lumbar spine flexion which accompanies hip flexion.

Three-dimensional measurement tools based on electromagnetic tracking have recently been used to precisely measure shoulder [10-12] and spine [13,14] ROM, as well as patellofemoral [15] and hip joint [16] kinematics. Electromagnetic tracking systems (ETS) enable the direct measurement of a three-dimensional position and the orientation of multiple sensors referred to a stationary source (transmitter). As ETS may well provide the reference standard to assess the ROM of the musculoskeletal system [17] in a clinical setting, concurrent use of ETS and simple two-dimensional measurement devices is a possible method to determine the validity of goniometers to yield plausible and useful objective ROM data [18-20].

Thus, the aims of this study were (i) to verify the validity of a conventional manual goniometer (i.e., the standard instrument for clinical assessments) to measure passive hip ROM against a criterion standard instrument (ETS) (concurrent validity) and to discriminate between individuals with and without FAI (known group construct validity), and (ii) to examine the test-retest (intra-rater) reliability of hip ROM goniometric and ETS assessments.

## Methods

### Subjects

A total of 15 subjects (7 women) with a diagnosis of FAI, verified both clinically and radiologically [3], were evaluated (age ± SD: 35 ± 11 years; height: 171 ± 8 cm; mass: 68 ± 8 kg). Patients were recruited if they were at least 18 years of age and treated at our institution (orthopedic hospital specialized in the treatment of the musculoskeletal system; core competencies include joint-preserving hip and knee surgery for mechanical malalignment and instability such as hip dysplasia and FAI). As a healthy control group, 15 sex- and age-matched adults (employees of our institution) volunteered to participate in the study (age: 34 ± 10 years; height: 173 ± 9 cm; mass: 68 ± 13 kg). For both subject groups, exclusion criteria included contraindications for ROM measurements and concomitant lower extremity injuries. All subjects provided written informed consent prior to data collection. Ethical clearance for the study was granted from the local Ethics Committee.

### Study design

Subjects were tested on two occasions, one week apart, at the same time of day and at constant room temperature to establish the test-retest reliability of the goniometer and the ETS for hip ROM assessment. Concurrent (criterion-based) validity was examined by simultaneously recording the same hip ROM movement with both devices, the ETS serving as the criterion instrument [17]. Known group construct validity was based on hip ROM recordings on both FAI patients and healthy controls, with between-group differences serving as the construct. Unilateral passive hip ROM for flexion, abduction, adduction, internal and external rotation (3 trials per movement, randomly presented) were assessed for both hips, and the initial side measured was randomized. A single investigator (SN; human movement scientist with 1 year of experience in musculoskeletal examination), blind to participants' characteristics, conducted all ROM movements. Two other co-investigators (SS, JFG; human movement scientists with 1 and 2 years of experience in musculoskeletal examination, respectively), both assisting in half of the testing sessions, were in charge of goniometric assessments (ETS values blinded). All investigators received additional training in goniometry and were instructed for reaching agreement on the method of measuring each hip movement by experienced physiotherapists and a hip orthopaedic surgeon. Up to three subjects were measured per single day, with a break of at least one hour in between.

### Experimental procedures

Prior to data collection, subjects completed a standardized warm-up consisting of ergometer cycling (5 minutes at 50 W). Then, each hip ROM movement was performed twice for further warm-up and familiarization. All assessments were performed in the supine position according to Clarkson [21], and sets of motion on the same plane were always measured consecutively (e.g., internal then external rotation). Hip flexion was measured with the knee fully flexed, abduction-adduction with the knee and hip fully extended and internal-external rotation with the hip and knee at 90°.

#### Goniometry

**Figure 1:**
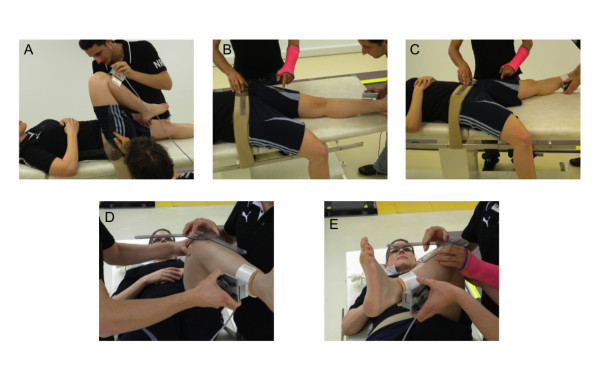
**Goniometric assessment of passive hip ROM**. A) Hip flexion. B) Hip adduction. C) Hip abduction. D) Hip internal rotation. E) Hip external rotation. Note the positions/roles of the two examiners, the alignment of the goniometer, and the position of the dynamometer pad.

A simple long-arm goniometer (Orthopedic Equipment Co., Bourbon, USA) with a 360° scale marked in one degree increments was used. Hip flexion was measured with the goniometer as the deviation from the neutral zero position in which the lower limb and trunk are in the horizontal plane (Figure 1A). The stationary arm of the goniometer was aligned over the horizontal axis of the body. The goniometer's moveable arm was aligned over the lateral midline of the thigh and the greater trochanter was used to center the fulcrum of the goniometer. Hip adduction (Figure 1B) and hip abduction (Figure 1C) were measured from the neutral zero position in which the longitudinal axis of the thigh is perpendicular to the transverse line across the anterior superior iliac spines of the pelvis. These latter anatomical landmarks were also used for alignment of the stationary arm of the goniometer. The unilateral anterior superior iliac spine was used to center the fulcrum of the goniometer and the moveable arm of the goniometer was aligned over the midline of the femur pointing at the center of the patella. The subject had the contralateral leg hanging down on the edge of the massage table to ensure that the pelvis was not moving during abduction and the leg was not constrained in performing adduction. For hip rotations, subjects had the hip and the knee flexed to 90°. The stationary arm of the goniometer was aligned parallel to the transverse line across the anterior superior iliac spines of the pelvis with the fulcrum of the goniometer centered over the patella apex. Internal rotation (Figure 1D) and external rotation (Figure 1E) were measured as the deviation from the zero starting position, in which the longitudinal axis of the leg was perpendicular to the transverse line across the anterior superior iliac spines.

#### ETS Protocol

**Figure 2:**
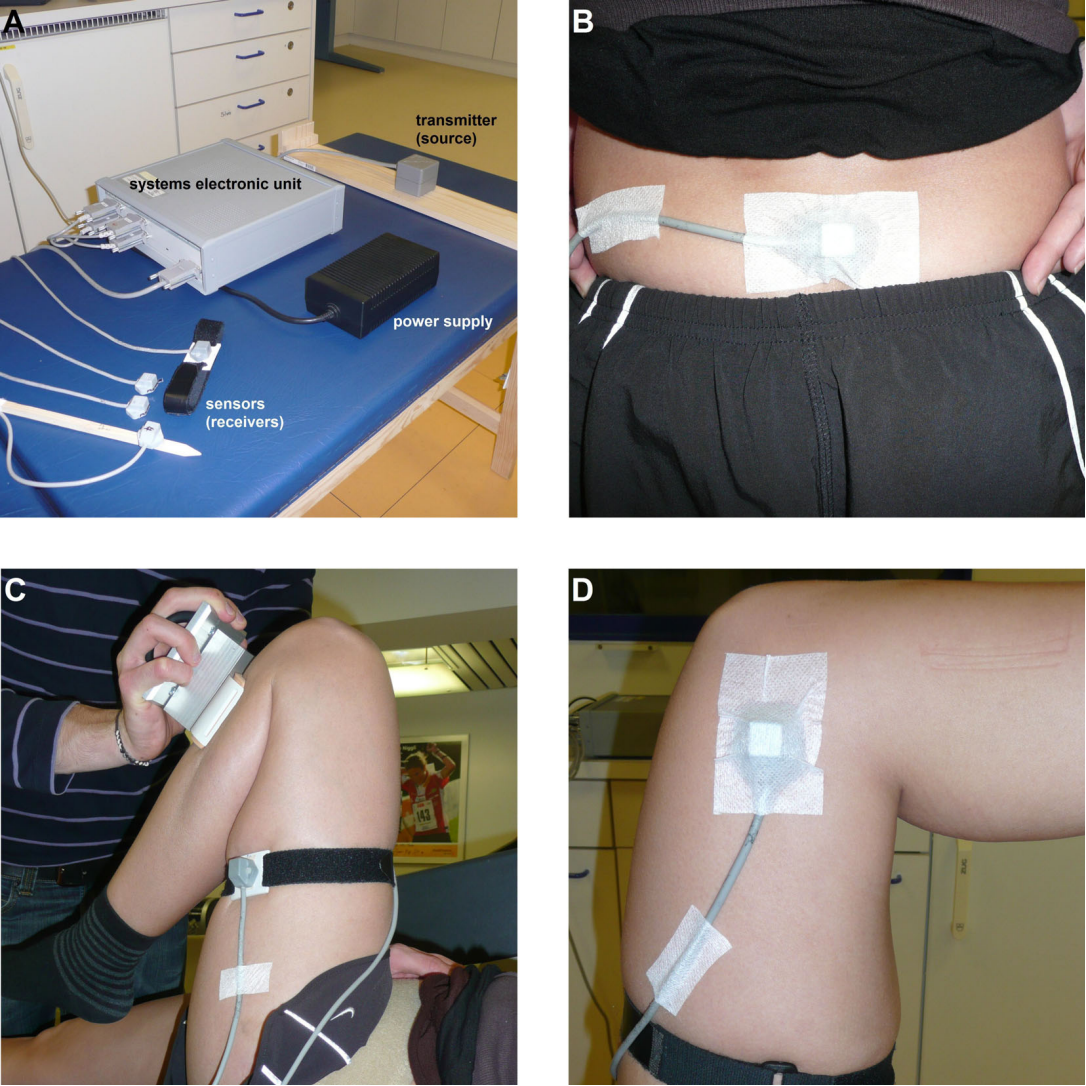
**Electromagnetic tracking system (ETS)**. A) ETS instrumentation. B) ETS sensor taped over the sacrum with double sided tape and medical adhesive tape. C) ETS sensor attached to a mouldable plastic plate and tightly wrapped around the lateral aspect of the thigh. Standardized force was applied by a modified hand-held load cell system. D) ETS sensor taped over the medial aspect of the knee with double sided tape and medical adhesive tape.

The ETS (Fastrak, Polhemus Inc., Colchester, USA), which consists of an electronic unit linked to a host computer, one transmitter and four sensors (Figure 2A), sampled data at 30 Hz. Measurements were performed on a self-made wooden massage table (200 × 60 × 66 cm) to avoid interference from metal objects, with the transmitter mounted on a wooden platform centrally attached under the massage table. Such configuration ensured that the operational range of the transmitter was sufficient for all movements. Pilot experiments showed that these settings allowed the ETS to work accurately in the capture volume of the study.

One sensor was attached to the sacrum with double-sided tape and flexible medical adhesive tape (5 × 10 cm), whilst the subject was in a standing position (Figure 2B). Subjects were then asked to lay supine with their back on foamed material (40 × 25 × 8 cm) with a cut-out, allowing the sacrum sensor to move freely. The body was stabilized by a belt around the pelvis, with the intent to constrain pelvic movements during data collection. Another sensor was attached to a moldable plastic plate with plastic screws and a Velcro band was threaded through the plate and tightly wrapped around the subject's thigh, so as to minimise the movement between the sensor and the underlying skin and to maximise coupling with the underlying skeletal features [16,22] (Figure 2C). The optimal sensor location to meet the above criteria was on the lateral side of the thigh, at mid distance between the lateral epicondyle of the femur and the greater trochanter. A third sensor was attached to the medial aspect of the knee, 2 cm proximal to the medial epicondyle of the femur (Figure 2D). The fourth sensor was embedded within a wooden calibration pointer to digitize palpated anatomical landmarks prior to data collection.

Anatomical and global calibration trials were performed with the subject in the supine position. The three-dimensional positions of several anatomical landmarks were located by sequentially placing the tip of the pointer on each landmark. Position and orientation data were subsequently sampled from both the calibration sensor and the sensor attached to the segment. Dynamic calibration trials were performed to enable the calculation of functional hip joint centers. This required the participants to perform two hip circumductions with a ROM of approximately 30° in flexion and 30° in abduction [23].

**Figure 3:**
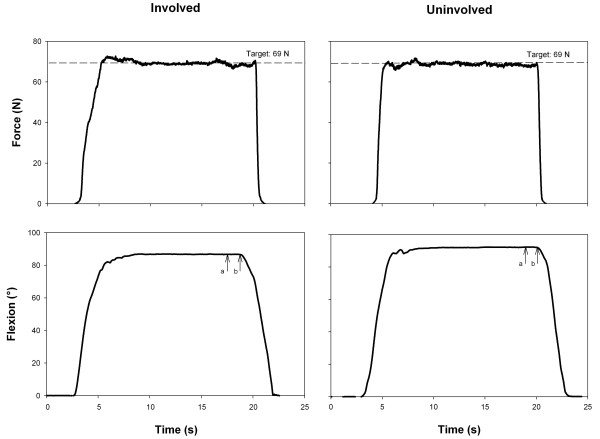
**Assessment of passive hip flexion**. Manually-applied force (top) and hip flexion range of motion (ROM) (bottom) traces of the involved and uninvolved side of a femoroacetabular impingement patient. The horizontal dotted lines indicate the target force (mean of the two warm-up trials). ROM was calculated as the mean angle during the 1-s interval between "a" and "b", where "b" is the greatest ROM. Note that ROM is greater for the uninvolved than for the involved side.

In order to further standardize hip joint ROM measurements, the force applied by the main investigator was controlled by a modified hand-held load cell system (Metitur, Jyvaskyla, Finland). Two plastic grips allowed the investigator to easily handle the load cell during the measurements. For hip flexion, the pad of the dynamometer was pressed against the shank, 5 cm distal to the tibial tuberosity (Figure 2C). For hip abduction (Figure 1C) and internal rotation (Figure 1D), the pad was applied 5 cm proximal to the medial malleolus, whereas it was positioned on the lateral aspect of the shank for hip adduction (Figure 1B) and external rotation (Figure 1E). For each movement, force was applied for 15 s by the investigator, who received consistent visual feedback of the applied force. The mean force of the two warm-up trials was used as the target force for both testing sessions (Figure 3, top traces). For each subject, the applied torque was calculated as the applied force multiplied by the lever arm. The torque was subsequently normalized to ROM (Nm/°). The mean normalized torque ± SD was 0.35 ± 0.14 Nm/° (FAI) and 0.38 ± 0.10 Nm/° (healthy) for flexion, 1.07 ± 0.29 Nm/° (FAI) and 1.20 ± 0.36 Nm/° (healthy) for abduction and adduction, and 0.69 ± 0.33 Nm/° (FAI) and 0.77 ± 0.39 Nm/° (healthy) for internal and external rotation. No significant differences were observed between sides and groups.

### Data processing

Data processing was performed using software written in the Matlab programming language (Mathworks Inc., Natick, USA). Functional hip joint centers, estimated using a functional approach, and digitized anatomical landmarks (medial and lateral epicondyle) were used to define the local coordinate systems of pelvis and thigh, which were then linked to the segment's individual receiver by means of coordinate transformations. For hip flexion, abduction and adduction, the sensor on the lateral aspect of the thigh was chosen as the relevant sensor to which the thigh coordinate system was related. As this sensor yielded considerably lower ROM for internal and external rotation compared to goniometry (-50% approximately), and also compared to the sensor on the medial aspect of the knee, the latter sensor served as the reference for these two motion patterns [24]. In general, the definitions of the local coordinate system for the pelvis and thigh segment followed the recommendations of the International Society of Biomechanics [25]. Once the orientations of the local coordinate systems for each segment were known, joint angles could be calculated using the floating axis method developed by Grood and Suntay [26]. The following sign convention was adopted: flexion, abduction and internal rotation were positive, while movements in the opposite directions (extension, adduction and external rotation) were represented by negative values. Figure 3 (bottom) shows typical ROM traces for flexion of the involved and uninvolved hips in a FAI patient. ROM was consistently calculated as the highest value over a 1-s interval. For each movement, the mean of the three trials was used.

### Statistical analysis

Data were first checked for normality and for homogeneity of variance. Paired Student t-tests were then used to detect any systematic bias between goniometer and ETS (concurrent validity) and test sessions (test-retest reliability). The Mann-Whitney U test was used to examine differences in ROM values between FAI patients and healthy controls (known group construct validity). For FAI patients, only the involved side was considered. For controls, the mean of the right and left hips was consistently used as no significant side-to-side difference was observed. Concurrent validity between the two systems was analyzed using intraclass correlation coefficients (ICC) (2,1) with their 95% confidence intervals, and Bland-Altman plots. This was done by plotting the difference between goniometer and ETS measures against their means and calculating the systematic bias ± random error, i.e., 95% limits of agreement (LOA) [27]. As proportional bias - i.e., a significant association between the difference of the two methods and the mean values - was observed for hip flexion and internal rotation by performing the Passing-Bablok regression analysis [28], LOA were also calculated according to the procedure proposed by Ludbrook [29]. This was done by constructing modified LOA lines running parallel to the predicted differences line of best fit, which can be used as an approximation of hyperbolic limits with increasing sample size [30]. Relative reliability, the degree to which individuals maintain their position in a sample with repeated measurements, was assessed using ICC (2,1) [31]. Absolute reliability, the degree to which repeated measurements vary for individuals, was analyzed using the coefficients of variation (CV), standard errors of measurement (SEM), and Bland-Altman plots (95% LOA) [31]. As a general rule, an ICC value over 0.75 was considered good [32]. In order to avoid statistical significance that might have occurred by chance, a corrected alpha level of *P *≤ 0.01 was accepted as significant (Bonferroni correction).

## Results

### Construct validity

For both measurement tools, hip abduction was significantly lower in FAI patients compared to controls (*P *< 0.01; Table 1), with group differences of 23% (ETS) and 24% (goniometer). Hip flexion, adduction, internal and external rotation as measured with both devices did not differ significantly between FAI patients and healthy controls, although a trend towards lower ROM was noted in the patient group.

**Table 1:** Passive hip ROM in patients with FAI and healthy subjects using goniometer and ETS

	Goniometer		ETS	
		
	FAI	Healthy	FAI	Healthy
Flexion (°)	103.8 ± 15.7	112.1 ± 11.3	84.5 ± 14.7	93.5 ± 7.8
Abduction (°)	30.4 ± 7.3	39.3 ± 7.4^a^	28.5 ± 6.7	37.3 ± 8.0^a^
Adduction (°)	23.2 ± 4.0	26.8 ± 5.7	21.5 ± 4.1	21.9 ± 3.0
Internal rotation (°)	26.0 ± 11.3	34.3 ± 10.1	24.2 ± 9.5	29.1 ± 8.5
External rotation (°)	36.3 ± 9.8	44.7 ± 4.8	29.6 ± 8.0	35.2 ± 4.2

Mean values ± SD. ROM: range of motion, FAI: femoroacetabular impingement, ETS: electromagnetic tracking system. ^a ^FAI lower than healthy, *P *< 0.01.

### Concurrent validity

**Figure 4:**
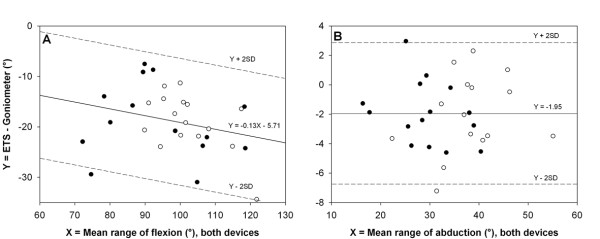
**Bland-Altman plots**. Comparison of the difference between the two methods of measurement (ETS and goniometer) versus the average of the two methods, for femoroacetabular impingement patients (•) and healthy subjects (_°_). Systematic bias is given by the solid line. Limits of agreement are given by the ± 2SD limits. A) Hip flexion. B) Hip abduction. Note that modified limits of agreement (with equations) are shown for hip flexion, as data revealed proportional bias.

Since ICC scores were comparable between FAI patients and controls, all concurrent validity analyses were completed for the whole group of subjects (n = 30). For the different ROM, ICC ranged between 0.44 and 0.94 (Table 2). The highest ICC was observed for hip abduction, and good validity was also observed for internal rotation; however, the low lower-bound confidence limit makes a meaningful interpretation of this latter ICC unwarranted. For hip flexion, adduction and external rotation, validity was not good. All ROM values were significantly greater for the goniometer compared to ETS (*P *< 0.001), therefore indicating systematic bias. The percentage difference between the two systems was 18% for flexion, 6% for abduction, 13% for adduction, 12% for internal rotation and 20% for external rotation. The highest systematic bias and random errors were observed for hip flexion ROM (Figure 4A, Table 2), while hip abduction showed the lowest bias and 95% LOA (Figure 4B, Table 2). No heteroscedasticity - i.e., a progressive increase of scatter of differences as the average increases [29] - was observed for all hip motion patterns.

**Table 2:** Concurrent validity of the goniometer with the ETS for hip ROM measurement in patients with FAI and healthy subjects

	ICC_2,1_	95% CI	LOA (°)
Flexion	0.440	-0.049 to 0.800	-18.92 ± 12.57^b^
Abduction	0.937	0.721 to 0.978	-1.95 ± 4.70^b^
Adduction	0.533	0.020 to 0.790	-3.32 ± 6.99^b^
Internal rotation	0.875	0.495 to 0.956	-3.50 ± 7.95^b^
External rotation	0.542	-0.087 to 0.844	-8.15 ± 8.49^b^

ETS: electromagnetic tracking system, ROM: range of motion, FAI: femoroacetabular impingement, ICC: intraclass correlation coefficient, CI: confidence interval

LOA: limits of agreement. ^b ^ETS lower than goniometer, P < 0.001. Modified LOA for data exhibiting proportional bias (x = mean value): (-0.13x - 5.71) ± 12.57 for hip flexion, (-0.22x - 2.65) ± 7.95 for internal rotation.

### Test-retest reliability

Test-retest ICCs were above 0.90 for all ROM assessments (Table 3), except for hip adduction (0.82-0.84). As a general observation, ICCs were quite similar between ETS and goniometer measures. In the same way, CVs (range 2.7-10.2%), SEMs (range 1.6-3.0°), and random errors (range 4.0-8.4°) for the ETS were very similar compared to the goniometer (range 3.1-7.7%, 2.4-3.9°, and 6.6-11.2°, respectively). Neither heteroscedasticity nor proportional bias was observed for the various motion patterns.

**Table 3:** Test-retest reliability of the goniometer and ETS for hip ROM measurement in patients with FAI and healthy subjects

	ICC_2,1_		CV (%)		SEM (°)		LOA (°)	
	
	Gonio	ETS	Gonio	ETS	Gonio	ETS	Gonio	ETS
Flexion	0.916	0.943	3.12	2.66	3.94	2.96	0.72 ± 11.15	0.27 ± 8.42

Abduction	0.924	0.947	5.84	5.66	2.36	2.01	0.14 ± 6.67	-0.08 ± 5.72

Adduction	0.842	0.823	6.73	6.34	2.36	1.59	-0.54 ± 6.60	1.07 ± 4.01

Internal rotation	0.950	0.902	7.74	10.19	2.42	2.93	-0.24 ± 6.89	1.32 ± 7.90

External rotation	0.914	0.934	5.23	5.10	2.53	1.81	0.02 ± 7.16	0.25 ± 5.14

ETS: electromagnetic tracking system, ROM: range of motion, FAI: femoroacetabular impingement, ICC: intraclass correlation coefficient, CV: coefficient of variation, SEM: standard error of measurement, LOA: limits of agreement, Gonio: goniometer. Note that bias (retest lower than test) was significant only for adduction measured with the ETS (*P *< 0.01).

## Discussion

This study examined whether manual goniometers (i) are sensitive enough to discriminate between individuals with and without FAI (construct validity), (ii) measure the anatomical correct hip joint ROM (concurrent validity) and (iii) produce consistent results (test-retest reliability). The major findings of this study were that goniometric measurements of passive hip motion provided greater ROM data than the criterion instrument ETS. Interestingly, the agreement between the two devices was high for hip abduction and internal rotation, but low for flexion, adduction, and external rotation. The finding that goniometers are particularly valid for measuring hip abduction was also confirmed by the comparison between FAI patients and healthy controls. Moreover, it was demonstrated that goniometric evaluation of passive hip joint angles was reliable between days, with similar reliability scores compared to the ETS. Manual goniometers can therefore be used with confidence during longitudinal assessments, which rely on repeated measurements over time.

The assessment of construct validity was performed by comparing hip ROM between FAI and healthy hips (known group validity). Considering the advanced number of FAI patients in our clinic and the quickly increasing interest for this pathology worldwide [3], information regarding limitation of hip ROM is needed. Due to significantly lower hip abduction ROM in the FAI group as measured with both devices, the current results demonstrated strong construct validity of manual goniometers for hip abduction assessment. The finding of lower abduction ROM in FAI patients is also supported by the literature [4,33,34]. For the other motion patterns, tendencies of lower ROMs in the FAI group were observed. However, the small sample size and heterogeneity in patient characteristics limit the interpretation of the present construct validity results. Comparisons to previous studies dealing with hip ROM differences between FAI and healthy hips are difficult because either no information about the measurement technique was provided [35-37] or a CT-based computer-assisted technique, ignoring cartilaginous structures, soft tissue contractures or masses for the calculation of ROM was applied [4]. Clohisy et al. [38] found no ROM differences between FAI and non-symptomatic hips, whereas Philippon et al. [34] reported significantly reduced ROM in injured hips for all directions. Although limited internal rotation in 90° of flexion seems to be the key symptom during clinical examination [4,35,39,40], the extent of restricted internal rotation still has to be ascertained as the results of this study did not reveal such a significant reduction of internal rotation in the FAI group compared to healthy controls. One possible explanation for this finding could be that in medical routine assessments, hip ROM examination is stopped before the passive limit is reached because of the groin pain that accompanies internal rotation due to sharing forces at the labrum. It is also possible that some subjects in the control group had abnormal bony anatomy, as the estimated prevalence of FAI ranges between 10 and 15% [40]. Adequately powered studies are needed to verify which ROM movements (together with hip abduction) should be included in physical examination as an indicator of FAI.

Although all hip ROM values measured by the goniometer were significantly greater compared to the ETS, concurrent validity of manual goniometers was particularly good for hip abduction, with high ICC, and low systematic bias and random error. Subjects were positioned supine with the contralateral leg hanging down on the edge of the massage table during hip abduction assessments. Thus, the pelvis was not only stabilized by the belt, but also by the abducted contralateral limb, regardless of any other movements. In this configuration, excessive rotation of the pelvis around a vertical axis was prevented, which was not the case for hip adduction. Considering that the sacrum sensor was assumed to be rigidly attached to the pelvis and therefore representative of the pelvic coordinate system, most of the differences between the goniometer and the ETS can be attributed to this phenomenon. For motion patterns others than hip abduction, it is plausible that the difference between the goniometer and the ETS was largely due to pelvic rotation when the passive limit of motion was reached. Although the co-investigators performing the goniometer assessments tried to minimize this source of error by stabilizing the subject manually, they could not adequately correct for this misalignment. The obtained results for hip flexion are in agreement with Elson and Aspinall [9], who found a mean value of 85° for true hip flexion. In the same way, Bohannon et al. [8] stated that a large portion of the hip flexion movement is assumed to be the consequence of pelvic rotation, resulting in pure hip flexion of only 90°. Independent of the arc of motion, pelvic tilt always occurred within the first 10° of hip flexion, indicating that the thigh and pelvis move in synergy with one another. Hence, for what we usually call hip flexion, the ROM generally evaluated is thigh flexion on the trunk, which is a combination of "true" hip flexion and pelvis tilt.

Apart from the uncontrolled pelvic tilt or rotation and neutralization of lumbar lordosis (in case of hip flexion) for goniometric hip ROM assessments, there are other possible factors leading to the disagreement between the goniometer and ETS data. It is unlikely that between-device bias was attributable to differences in the physiological mechanisms underlying hip ROM testing, because measurements were performed simultaneously with the two devices. Rather, the observed discrepancies were certainly due to visual estimation of the true anatomical reference lines (e.g., the long axis of the limb) and potential alignment of the goniometer to the position of the massage table or to the laboratory arrangements, rather than to true bony orientation. Another reason for the disagreement between the goniometer and ETS is the two-dimensional characteristics of goniometric hip ROM measurements. Cheng and Pearcy [41] showed that an abduction angle measured in the frontal plane and a flexion angle measured in the sagittal plane can be significantly overestimated by the presence of out-of-plane movements. In the setting of this study, both hip abduction and adduction were associated to some degree of flexion, whereas hip flexion was associated to some degree of abduction. Therefore, this kind of error may have occurred, at least to a small extent.

The results obtained in the present study suggest that the hip ROMs "read" by the goniometer are in fact intersegmental thigh-trunk angles (e.g., thigh flexion on trunk for hip flexion) rather than true hip joint ROM. This study clearly demonstrates the "harmful" effect of flexing hip while evaluating ROM if pelvic tilt or rotation is not adequately controlled. Future research is needed to find hints allowing the goniometer to measure an angle close to the real ROM and to verify if other ways of goniometric estimation (e.g., hip rotations measured in prone position) would provide similar results compared to the ETS, all the more because one of the goniometer's arms could be placed on the table, potentially adding accuracy due to standardization. Even if manual goniometers are logically preferred in the clinic to more accurate devices, such as ETS, because of the limited time available for routine medical examinations, clinicians should be aware of this misinterpretation and they should try to minimize pelvic rotation. Apart from other advantages (such as simplicity of use, low cost and time saving), the use of conventional manual goniometers for longitudinal evaluations is supported by the present test-retest reliability results, which were good and comparable to those obtained with the ETS.

The excellent absolute and relative reliability estimates for hip flexion are in agreement to those reported on persons with hip osteoarthritis [1,2,42-44] and healthy hip subjects [5,45]. Results from CV analyses showed that hip flexion measurements had the lowest difference between the two test days. ROM measurements of the hip abductors showed excellent test-retest reliability, with CVs around 5% and ICC estimates exceeding 0.90. A review of the literature did not identify reliability studies with comparable good CV results. Holm et al. [2] and Pua et al. [44] both reported CVs of more than 20% for passive hip ROM measurements. Even in a study assessing intra-tester within session reliability [5], CV was larger compared to the present between session results. The excellent reliability estimates for hip abduction are the result of adequate stabilization of the subject's body and standardized force. Of particular interest in this study was the measurement of hip adduction ROM, as information about the test-retest reliability of this movement pattern is lacking in the literature. Those authors providing information about the repeatability of hip adduction measurements reported CVs of 23% [2], ICCs around 0.5 [2,43,46], or used the Pearson correlation coefficient, which is a questionable reliability index [1]. Although superior, the results of this study indicate that hip adduction is the most challenging movement pattern to measure, confirmed by the low ICCs (< 0.9) and the smallest arc of motion (~20°). The ICC estimates for hip internal and external rotation (~0.9) are in agreement with those previously reported on hip osteoarthritis subjects [2,44]. In contrast, Croft et al. [42] reported substantially lower levels of reliability by using six raters and six participants with hip osteoarthritis. They reported inter-tester ICC values of 0.48 and 0.43 for internal and external rotation, respectively. However, because of their small sample size, the results must be interpreted with caution. The present CV values are even slightly superior to those reported in the literature [2,5,44]. Nevertheless, inter-study comparisons for rotational movements are difficult because of differences in the testing positions (prone compared to supine).

The current study has a few limitations. Firstly, human movement analysis based on electromagnetic tracking technology is affected by instrument errors, anatomical landmark uncertainty and skin movement artifacts [47]. These sources of error were minimized by using a functional approach for calculating the hip joint centers and by adopting a segment coordinate system approach, which reduced anatomical landmark palpation to a minimum. However, it is still unknown if the joint coordinate system solution proposed by Grood and Suntay [26] represents the clinical reality. Moreover, movements were primarily performed in a single plane, so that error due to inertial effects and skin deformation by direction of movement were both negligible. The authors therefore believe the ETS can be used as the criterion instrument for hip joint ROM assessment in orthopedic research, although it is acknowledged that alternative methods such as fluoroscopy or bone-pins would be more accurate. Secondly, ETS measurements were limited to a single investigator performing the movements. Future research should include more testers in order to determine inter-tester reliability. It can be supposed that this kind of reliability would also yield excellent results for both devices, as standardization of the applied force should eliminate a considerable amount of inter-tester variability [5,48,49]. Finally, the investigator performing the goniometer assessments was not blinded for the goniometric ROM values. This bias was minimized by prohibiting the observer to read the goniometer results until proper alignment of the device was ensured. It is therefore unlikely that this factor influenced the main results.

## Conclusions

This study was designed to determine the validity and reliability of manual goniometers for measuring passive hip ROM. This study is unique to the literature, and thus offers new information of clinical importance. The current findings suggest that goniometer-based assessments conventionally used in orthopedic clinical practice overestimate the majority of passive hip motion patterns by measuring intersegmental angles (e.g., thigh flexion on trunk for hip flexion) rather than true hip ROM; it is indeed difficult to reproduce true hip ROM by placing the goniometer properly and performing the anatomically correct ROM. It is likely that uncontrolled pelvic rotation and tilt contribute to the overrating of the arc of these motions. Future work is needed to find hints to perform hip ROM assessments more accurately. It is concluded that, as manual goniometers yielded good test-retest reliability estimates, they would remain the first choice tool for the assessment of hip ROM in the clinic, especially when longitudinal monitoring of hip function is aimed.

## Competing interests

The authors declare that they have no competing interests.

## Authors' contributions

SN, NAM, ML and HG were responsible for the conception and design of the study. SN, JFG and SS coordinated the practical work and performed data acquisition. SN prepared the data for analysis and performed together with NAM the statistical analyses, data interpretation and manuscript drafting. ML, HG, JFG and SS assisted with some of the statistical analyses and with the interpretation of the data. All authors read and approved the final manuscript.

## Pre-publication history

The pre-publication history for this paper can be accessed here:

http://www.biomedcentral.com/1471-2474/11/194/prepub
